# Differential effects of TRPM4 channel inhibitors on Guinea pig urinary bladder smooth muscle excitability and contractility: Novel 4‐chloro‐2‐[2‐(2‐chloro‐phenoxy)‐acetylamino]‐benzoic acid (CBA) versus classical 9‐phenanthrol

**DOI:** 10.1002/prp2.982

**Published:** 2022-07-13

**Authors:** John Malysz, Sarah E. Maxwell, Georgi V. Petkov

**Affiliations:** ^1^ Department of Pharmaceutical Sciences, College of Pharmacy University of Tennessee Health Science Center Memphis Tennessee USA; ^2^ Present address: Department of Physiology and Cell Biology University of Nevada Reno Nevada USA

**Keywords:** bladder, contraction coupling, patch clamp, smooth muscle, transient receptor potential channels

## Abstract

Non‐selective cation channels in urinary bladder smooth muscle (UBSM) are thought to mediate increases in cellular excitability and contractility. For transient receptor potential melastatin type‐4 (TRPM4) channels, the evidence primarily relies on the inhibitor 9‐phenanthrol, which exhibits pharmacological limitations. Recently, 4‐chloro‐2‐[2‐(2‐chloro‐phenoxy)‐acetylamino]‐benzoic acid (CBA) has been discovered as a novel TRPM4 channel blocker. We examined how, in comparison to 9‐phenanthrol, CBA affects the excitability of freshly isolated guinea pig UBSM cells and the contractility of UBSM strips. Additionally, non‐selective TRPM4 channel inhibitor flufenamic acid (FFA) and potentiator BTP2 (also known as YM‐58483) were studied in UBSM cells. Unlike robust inhibition for 9‐phenanthrol already known, CBA (up to 100 μM) displayed either no or a very weak reduction (<20%) in spontaneous phasic, 20 mM KCl‐induced, and electrical field stimulated contractions. For 300 μM CBA, reductions were higher except for an increase in the frequency of KCl‐induced contractions. In UBSM cells, examined under amphotericin B‐perforated patch‐clamp, CBA (30 μM) did not affect the membrane potential (*I* = 0) or voltage step‐induced whole‐cell cation currents, sensitive to 9‐phenanthrol. The currents were not inhibited by FFA (100 μM), increased by BTP2 (10 μM), nor enhanced under a strongly depolarizing holding voltage of −16 or + 6 mV (vs. −74 mV). None of the three compounds affected the cell capacitance, unlike 9‐phenanthrol. In summary, the novel inhibitor CBA and nonselective FFA did not mimic the inhibitory properties of 9‐phenanthrol on UBSM function. These results suggest that TRPM4 channels, although expressed in UBSM, play a distinct role rather than direct regulation of excitability and contractility.

## Graphical abstract

The novel TRPM4 channel inhibitor CBA does not mimic the inhibitory effects of 9‐phenanthrol on guinea pig USBM contractility and excitability implying a distinct regulatory role of TRPM4 channels in the urinary bladder function.

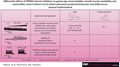

**Abbreviations**
BTP2: N‐[4‐[3,5‐Bis(trifluoromethyl)pyrazol‐1‐yl]phenyl]‐4‐methylthiadiazole‐5‐carboxamide, alias YM‐58483
Ca_V_
: voltage‐gated Ca^2+^ (channel)
CBA: 4‐chloro‐2‐[2‐(2‐chloro‐phenoxy)‐acetylamino]‐benzoic acid
ddH_2_0: double distilled H_2_0
EFS: electrical field stimulation
FFA: flufenamic acid
IK: intermediate conductance calcium activated K^+^ (channel)
K_Ca_1.1: voltage and calcium activated large conductance K^+^ (channel), alias BK or MaxiK^+^
K_V_
: voltage‐gated K^+^ (channel)
Na_V_
: voltage‐gated Na^+^ (channel)
UBSM: urinary bladder smooth muscle
TRPM4: transient receptor potential melastatin type‐4

## 1 INTRODUCTION

Urinary bladder smooth muscle (UBSM) relaxation and contraction determine urine storage or voiding, respectively.^1^ UBSM cells express various types of ion channels that either inhibit (e.g., voltage‐dependent K^+^ channel type 2 or K_V_2, K_V_7, and K_Ca_1.1) or promote (e.g., voltage‐gated Ca^2+^ channel type 1.2 or Ca_V_1.2 and Ca_V_3) cell excitability.^2^ Seminal studies on guinea pigs^3^ and rats^4^ and later on humans^5^ and mice^6^, ^7^ identified the transient receptor potential melastatin type‐4 (TRPM4) channel as a likely regulator of UBSM excitation‐contraction coupling.

Unlike most TRP channels, TRPM4 channels exhibit high selective permeability to only monovalent cations (Na^+^ and K^+^) but not to divalent cations like Ca^2+^ and Mg^2+^, or anions.^8^, ^9^, ^10^ Intracellular Ca^2+^ activates TRPM4 channels, whereas ATP inhibits them, thus linking intracellular signaling and metabolism to channel regulation.^8^, ^9^, ^10^ In the case of smooth muscle cells including UBSM cells, the opening of cell membrane TRPM4 channels is thought to induce depolarization, causing subsequent opening of Ca_V_ channels, net Ca^2+^ influx, increase in intracellular Ca^2+^, and activation of contractile machinery.^3^, ^4^, ^5^, ^11^, ^12^

Two major lines of evidence have been provided for the role of TRPM4 channels in UBSM function. First, UBSM tissues and cells showed robust expression of TRPM4 mRNA and protein.^3^, ^4^, ^5^, ^7^, ^12^, ^13^ Second, 9‐phenanthrol, a widely used TRPM4 channel inhibitor,^14^ caused attenuation of UBSM cell excitability and inhibition of contractility in UBSM strips.^3^, ^4^, ^5^, ^6^, ^7^, ^12^, ^13^, ^15^ The presence of TRPM4 channels and pharmacological studies using TRPM4 channel modulators, especially 9‐phenanthrol, have also revealed a role of TRPM4 channels in insulin secretion (pancreatic β‐cells), cardiac excitability (ventricular cells), immune response (lymphocytes and monocytes), and vascular smooth muscle excitability and contractility.^10^, ^11^, ^16^, ^17^, ^18^ 9‐Phenanthrol, hence, has been a key compound in investigating TRPM4 channels in various cell types.^14^

Pharmacologically, 9‐phenanthrol shows modest potency (IC_50_) of ∼15–30 μM for TRPM4 channels expressed in human embryonic kidney (HEK)‐293 cells determined electrophysiologically or with an imaging assay utilizing a Na^+^‐sensitive dye.^19^, ^20^ In experiments on native cells, the tested concentration is typically in the range of 10–100 μM. In UBSM cells, the most effective concentration is 100 μM with some non‐specific effects most likely occurring due to loss of cell viability at higher concentrations.^15^ Selectivity of 9‐phenanthrol for TRPM4 channels is not optimum. When tested at 10–100 μM, 9‐phenanthrol has been reported to also block TMEM16A/Ano1 Cl^−^, Ca_V_ , Na_V_ _,_ K_V_ , and IK currents and to induce cytotoxicity.^21^, ^22^, ^23^, ^24^, ^25^ The limitations of 9‐phenanthrol including its poor aqueous solubility have led to the need of discovering more selective TRPM4 channel inhibitors. One such compound recently identified is CBA. CBA inhibits TRPM4 channels with a 20‐fold greater potency than 9‐phenanthrol.^20^ Other modulators, albeit non‐selective, used to study TRPM4 channels are flufenamic acid (FFA) and BTP2 (also known as YM‐58483), which, respectively, block and potentiate them.^20^, ^26^, ^27^

In this study, we aimed to further corroborate the role of TRPM4 channels in UBSM excitability and contractility using the novel TRPM4 channel inhibitor, CBA, and two other known modulators, the activator BTP2 and inhibitor FFA, in guinea pig UBSM. We found unexpectedly that CBA and FFA did not mimic the inhibitory effect of 9‐phenanthrol nor did we observe any change for BTP2 on UBSM excitability. We elaborate on the implications of these novel findings regarding TRPM4 channel function in UBSM.

## 2 MATERIALS AND METHODS

### 2.1 UBSM tissue harvesting

Experiments were conducted in accordance with the Animal Use Protocols No. 17‐075 and 20‐019 reviewed and approved by the University of Tennessee Health Science Center (Memphis, TN). For this study, 31 adult male Hartley‐Albino guinea pigs (median weight: 662 g, 25th percentile: 538 g; 75th percentile: 931 g) were euthanized either with CO_2_ or isoflurane (Forane^®^, Baxter, Deerfield, IL) followed by thoracotomy. Then, the whole bladder was cut above the bladder neck and transferred to a Petri dish containing dissection/digestion solution (see *Solutions and compounds* section for composition). The whole bladder was excised, and the mucosa including urothelium removed. UBSM strips (5–10 mm long and 2–4 mm wide) were prepared for isometric tension recordings and for UBSM single‐cell isolation.

### 2.2 Fresh enzymatic single UBSM cell isolation

Guinea pig UBSM cells were isolated as previously described.^3^, ^15^ Briefly, small UBSM strips were cut from the mucosa‐free UBSM specimens and placed in dissection/digestion solution (1–2 ml) supplemented with 1 mg/ml bovine serum albumin (BSA), 1–1.5 mg/mL papain, and 1 mg/mL dithiothreitol and incubated for 15–35 min at ~37°C. Next, UBSM strips were transferred to dissection solution (1–2 ml) containing 1 mg/mL BSA, 1–2 mg/mL type II collagenase, and 100–200 μM CaCl_2_ for 15–35 min at ~37°C. After the incubation, UBSM strips were washed with fresh dissection/digestion solution containing BSA. Individual UBSM cells were released from the tissue by passing through a Pasteur pipette. UBSM cells were immediately used in electrophysiological patch‐clamp recordings or stored at ~4°C for further use within 6 h.

### 2.3 Electrophysiological experiments

Amphotericin B‐perforated whole‐cell patch‐clamp recordings were performed based on methods and procedures previously described.^3^, ^15^, ^28^ Briefly, several drops of the dissection/digestion solution containing freshly isolated UBSM cells were placed into a recording chamber. After at least 45 min, the cells were washed several times with the extracellular (bath) solution (see *Solutions and compounds*). Voltage step‐induced currents or membrane potentials (current clamp, *I* = 0) were recorded using an Axopatch 200B amplifier, Digidata 1440A, and pCLAMP version 10 software (Molecular Devices, Union City, CA) and solutions optimized for the recording of cation currents (K^+^ currents minimized, see *Solutions and compounds*). The patch‐clamp pipettes were prepared from borosilicate glass (Sutter Instruments, Novato, CA) and pulled using a Sutter P‐97 horizontal puller (Sutter Instrument Co., Novato, CA). Pipettes were fire‐polished with a Microforge MF‐830 (Narishige Group, Tokyo, Japan) to give a final tip resistance of approximately 3–6 MΩ. In perforated voltage‐clamp patch‐clamp experiments, UBSM cells were held at −74 mV (corrected for the junction potential), voltage‐stepped from −94 to 106 mV (also corrected for the junction potential) in 10 mV increments for 400 or 500 ms, and returned to −74 mV. Membrane potential recordings were obtained using the current‐clamp (*I* = 0) mode and physiological (high Na^+^ and Cl^−^ in bath and lowered intracellular/pipette Cl^−^) solutions (see *Solutions and compounds* section); the membrane potential values were corrected for the junction potential. All experiments were carried out at room temperature (21–23°C) within the same day of UBSM cell isolation. The maximum concentration of DMSO vehicle to dissolve CBA and 9‐phenanthrol did not exceed 0.2%. At this concentration, DMSO does not affect UBSM function.^29^, ^30^

### 2.4 Isometric UBSM tension recordings

The isometric UBSM tension recording experiments were conducted as previously described.^3^, ^13^, ^15^ Briefly, UBSM strips were clipped between a stationary mount and a force‐displacement transducer and then placed in tissue baths filled with Ca^2+^‐containing physiological salt solution (PSS) thermostatically controlled at 37°C. UBSM strips were stretched to an initial level of ~10 mN (1 g) and washed with fresh PSS every 15 min for 45–60 min equilibration period. For spontaneous and 20 mM KCl‐induced phasic contractions (obtained by supplying an additional 15.3 mM KCl to bath), the neuronal Na^+^ channel blocker tetrodotoxin (1 μM) was then added to the baths to attenuate neuronal activity. Once stable contractions developed (following at least 1 h equilibration period in the presence of tetrodotoxin) increasing concentrations of CBA were applied cumulatively at 10 to 12 min intervals on spontaneous or 20 mM KCl‐induced phasic contractions. In separate experiments, nerve‐evoked UBSM contractions were induced by electrical field stimulation (EFS) using a pair of platinum electrodes mounted in the tissue bath parallel to the UBSM strip. The EFS pulses were generated using a PHM‐152I stimulator (Med Associates, Georgia, VT). The EFS pulse parameters were as follows: 0.75‐ms width, 20‐V amplitude, and 3‐s stimulus duration, with polarity reversed for alternating pulses. For EFS studies, after the equilibration period, UBSM strips were subjected to continuous stimulation with a 10 Hz stimulation frequency at 1 min intervals and increasing concentrations of CBA were applied cumulatively at 10 to 12 min intervals. Unlike spontaneous and 20 mM KCl‐induced phasic contractions, all EFS responses were recorded in the absence of tetrodotoxin (1 μM).

### 2.5 Solutions and compounds

The dissection/digestion solution with or without supplemented Ca^2+^ (100–200 μM) had the following composition (in mM): 80 monosodium glutamate, 55 NaCl, 6 KCl, 10 glucose, 10 HEPES, and 2 MgCl_2_, pH 7.3, adjusted with NaOH. The Ca^2+^‐containing PSS for UBSM contractility studies was freshly prepared daily and contained the following (in mM): 119 NaCl, 4.7 KCl, 24 NaHCO_3_, 1.2 KH_2_PO_4_, 2.5 CaCl_2_, 1.2 MgCl_2_, 11 glucose, and aerated with 95% O_2_‐5% CO_2_ to obtain pH 7.4. In perforated voltage‐clamp experiments to measure the voltage step‐induced cation currents, the extracellular (bath) solution contained the following (in mM): 10 tetraethylammonium‐chloride (TEA‐Cl), 6 CsCl, 124 NaCl, 1 MgCl_2_, 2 CaCl_2_, 10 4‐(2‐hydroxyethyl)‐1‐piperazineethanesulfonic acid (HEPES), 10 glucose, and 0.002–0.003 nifedipine (pH 7.4 adjusted with CsOH), and pipette solution supplemented with freshly dissolved 300–450 μg/ml amphotericin B (in mM): 110 CsOH, 110 aspartic acid, 10 NaCl, 1 MgCl_2_, 10 HEPES, 0.05 ethylene glycol‐bis(β‐aminoethyl ether)‐N,N,N′,N′‐tetraacetic acid (EGTA), and 30 CsCl (pH 7.2 adjusted with CsOH). Membrane potential recordings were made using the amphotericin B‐perforated current‐clamp (*I* = 0) in the presence of the following extracellular solution (in mM): 134 NaCl, 6 KCl, 1 MgCl_2_, 2 CaCl_2_, 10 glucose, and 10 HEPES, pH adjusted to 7.4 with NaOH; and intracellular pipette solution containing freshly dissolved 300–450 μg/mL amphotericin B (in mM): 110 potassium aspartate, 30 KCl, 10 NaCl, 1 MgCl_2_, 10 HEPES, and 0.05 EGTA, pH adjusted to 7.2 with NaOH. Papain and collagenase type II were bought from Worthington (Lakewood, NJ) or Sigma‐Aldrich/Millipore (St. Louis, MO). CBA (cat no: 0SSK_529112) was purchased from Princeton BioMolecular Research, flufenamic acid from Fluka Analytical (Honeywell Fluka™, Charlotte, NC), and BTP2 from Cayman Chemical (Ann Arbor, MI). All other chemicals were obtained from Sigma‐Aldrich/Millipore or Fisher Scientific (Pittsburgh, PA). Stock solutions of CBA (30 or 100 mM) and nifedipine (10 mM) were prepared in DMSO. Tetrodotoxin (citrate) was dissolved in ddH_2_0 at 1 mM stock. Compounds were diluted in extracellular solutions and tested at concentrations as specified.

### 2.6 Data analyses and statistics

Clampfit software version 10, GraphPad Prism version 8.0 (La Jolla, CA), and Microsoft Excel were used for the analysis of patch‐clamp data. Steady‐state cation currents for the pre‐application controls, in the presence of a test condition either ion substitution or compound addition, and wash‐out (if applicable) were analyzed as averages over the last 200 ms of each voltage step. The average current values were then divided by the cell capacitance (constantly monitored) to calculate the current densities at each voltage. The responses for all voltages were then normalized to the current density measured at +106 mV for the pre‐application control of each cell unless stated otherwise. Analyses determining the effects of compounds on currents were carried out by subtracting the responses (current density values) obtained for a given compound at a voltage step from that of the pre‐addition and normalized to the pre‐addition control of +106 mV for each cell. Cell capacitance was constantly monitored during the course of the recording. In the case of membrane potential recordings, 3 min intervals prior to and after the addition of CBA for at least 5 min were analyzed as averaged values. Isometric UBSM tension recordings were obtained live with MyoMed software (Med Associates, St. Albans, VT). MiniAnalysis software (Synaptosoft, Decatur, GA) was used for data analysis of UBSM phasic contraction amplitude, muscle force integral (determined by integrating the area under the curve of the phasic contractions), frequency, duration (determined at half‐width level), and muscle tone over the last 5 min interval before the addition of a higher concentration of CBA. GraphPad Prism 8.0 software (GraphPad Software, La Jolla, CA) or Excel (Microsoft) were used for statistical analyses (including the testing for normal data distribution, ANOVA with a mixed model if applicable, and Student's t‐tests), and CorelDraw Graphic Suite software (Corel, Ottawa, ON, Canada) or Microsoft PowerPoint (Seattle, WA) for data illustration. The data are summarized as means ± SEM for normally distributed data or median (25th–75th percentiles) for non‐normal datasets, *n* = number of UBSM cells or tissue strips; *N* = number of guinea pigs used. Data (n values) were compared using two‐way ANOVA and paired or unpaired Student's *t*‐test as appropriate. A *p* value <.05 (two‐tailed for all) was considered statistically significant.

### 2.7 Nomenclature of targets and ligands

Key protein targets and ligands in this article are hyperlinked to corresponding entries in http://www.guidetopharmacology.org, the common portal for data from the IUPHAR/BPS Guide to PHARMACOLOGY,^31^ and are permanently archived in the Concise Guide to PHARMACOLOGY 2019/20.^32^

## 3 RESULTS

### 3.1 Differential effects of CBA and 9‐phenanthrol on UBSM spontaneous phasic, 20 mM KCl‐induced phasic, and EFS‐evoked contractions

**FIGURE 1:**
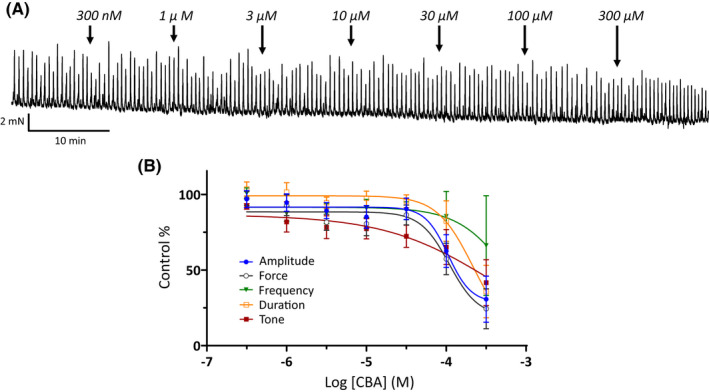
Modest efficacy of the novel TRPM4 channel inhibitor CBA on spontaneous phasic UBSM contractions. (A) Shown is a representative isometric UBSM tension recording demonstrating the effect of cumulative additions of CBA (300 nM to 300 μM) on spontaneous phasic UBSM contractions. (B) Presented are summary concentration‐response graphs for CBA on UBSM phasic contraction amplitude, muscle force, duration (half‐width), frequency of spontaneous phasic contractions, and muscle tone (*n* = 9, *N* = 8).

**FIGURE 2:**
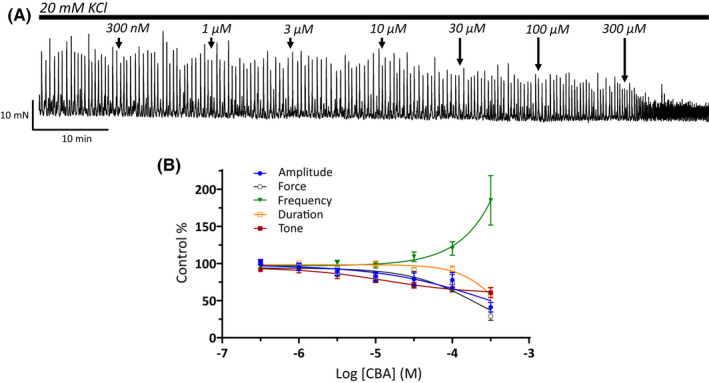
Weak inhibitory effects of CBA on 20 mM KCl‐induced phasic UBSM contractions. (A) Shown is a representative isometric UBSM tension recording demonstrating the effect of cumulative additions of CBA (300 nM–300 μM) on 20 mM KCl‐induced phasic UBSM contractions. (B) Presented are summary concentration‐response graphs for CBA on UBSM phasic contraction amplitude, muscle force, duration (half‐width), frequency of 20 mM KCl‐induced phasic contractions, and muscle tone (*n* = 17, *N* = 11).

**FIGURE 3:**
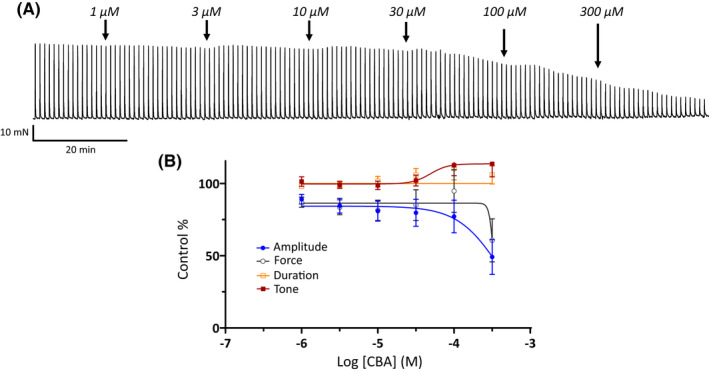
Weak potency of CBA on nerve‐evoked UBSM contractions. (A) Shown is a representative isometric UBSM tension recording demonstrating the effect of cumulative additions of CBA (1–300 μM) on nerve‐evoked UBSM contractions induced by continuous EFS with 10 Hz stimulation at 1 min intervals. (B) Presented are summary concentration‐response graphs for CBA on UBSM nerve‐evoked contraction amplitude, muscle force, duration (half‐width), and muscle tone (*n* = 11, *N* = 4).

We have previously shown that 9‐phenanthrol, a classical TRPM4 channel blocker, effectively inhibited guinea pig UBSM contractility^3^, ^15^; see also Tables 1, 2, 3. Here, we further examined how the novel TRPM4 channel inhibitor CBA affects UBSM contractility in the same animal species. The effects of CBA were examined on spontaneous phasic (Figure 1 and Table 1), 20 mM KCl‐induced (Figure 2 and Table 2), and EFS‐evoked contractions (Figure 3 and Table 3) over a range of increasing concentrations from 300 nM up to 300 μM. For all phasic contraction parameters (amplitude, force, duration, and/or frequency) CBA did not attenuate contractility at concentrations up to and including 30 μM. At the highest tested concentrations of CBA, inhibitory effects were observed. For spontaneous phasic contractions, the contraction amplitude and muscle force were maximally decreased by 70%–75% with IC_50_ values in the range of 130–160 μM (Figure 1 and Table 1). Comparable maximum inhibitions were observed for 20 mM KCl‐induced contractions with 60%–70% inhibition of both contraction amplitude and muscle force with IC_50_ values ranging from 200 to 300 μM (Table 2). The amplitude and muscle force of EFS‐evoked contractions exhibited maximum inhibitions of 40%–50% with IC_50_ values >300 μM (Figure 3 and Table 3). Of note, the frequency for 20 mM KCl‐induced phasic contractions, unlike spontaneous phasic and EFS‐evoked contractions, increased at the highest concentrations tested (Figure 2A,B and Table 2). The comparison of responses to CBA and 9‐phenanthrol on the three types of contractions revealed that the effects of the former compound were substantially weaker (Tables 1, 2, 3). The absence of a robust inhibitory effect of CBA on UBSM contractility was surprising given the compound was reported to have ~20‐fold higher potency and comparable maximum efficacy to 9‐phenanthrol for TRPM4 channels.^20^

**TABLE 1:** Summary of potency and maximum efficacy values for CBA and 9‐phenanthrol on UBSM spontaneous phasic contraction parameters and muscle tone

Contraction parameter	CBA	9‐Phenanthrol^a^
	IC_50_, Mean (95% CI) Max inhibition, Mean ± SEM (*n* = 4–9, *N* = 4–8)	IC_50_, Mean (95% CI) Max inhibition, Mean ± SEM (*n* = 12, *N* = 10)
Amplitude	163.8 (95.2–281.7) μM 69.3 ± 15.3%	1.5 (0.4–6.3) μM 73.3 ± 11.5%
Force	130.9 (69.7–245.7) μM 75.6 ± 13.2%	1.1 (0.4–3.2) μM 78.3 ± 9.4%
Duration (Half‐Width)	230.2 (161.1–328.9) μM 64.2 ± 17.4%	6.7 (1.4–15.3) μM 70.7 ± 12.7%
Frequency	> 300 μM 33.8 ± 32.9%	10.0 (2.6–49.7) μM 76.4 ± 10.2%
Muscle Tone	339.1 (62.7–1833) μM 58.3 ± 15.2%	1.3 (0.4–4.7) μM 35.1 ± 6.9%

^a^ Data for 9‐phenanthrol are from Malysz et al.^15^

**TABLE 2:** Summary of potency and maximum efficacy values for CBA and 9‐phenanthrol on 20 mM KCl‐induced UBSM phasic contraction parameters and muscle tone

Contraction parameter	CBA	9‐Phenanthrol^a^
	IC_50_, Mean (95% CI) Max inhibition, Mean ± SEM (*n* = 10–17, *N* = 6–11)	IC_50_, Mean (95% CI) Max inhibition, Mean ± SEM (*n* = 12, *N* = 10)
Amplitude	302 (138.7–657.9) μM 58.9 ± 6.4%	8.7 (3.7–20.0) μM 75.7 ± 6.8%
Force	191.9 (97.6–377.4) μM 70.9 ± 5.6%	5.8 (2.7–7.9) μM 81.5 ± 5.0%
Duration (Half‐Width)	> 300 μM 42.4 ± 10.4%	21.9 (6.3–75.9) μM 62.0 ± 8.6%
Frequency	> 300 μM −85.1 ± 33.4%^b^	13.3 (4.0–44.4) μM 64.0 ± 10.4%
Muscle tone	> 300 μM 9.3 ± 6.5%	3.4 (0.8–13.9) μM 38.7 ± 6.4%

^a^ Data for 9‐phenanthrol are from Malysz et al.^15^

^b^ Negative response indicates an increase in contractility.

**TABLE 3:** Summary of potency and maximum efficacy values for CBA and 9‐phenanthrol on nerve‐evoked UBSM contraction parameters

Contraction parameters	CBA	9‐Phenanthrol^a^
	IC_50_, Mean (95% CI) Max inhibition, Mean ± SEM (*n* = 10, *N* = 4)	IC_50_, Mean (95% CI) Max inhibition, Mean ± SEM (*n* = 12, *N* = 8)
Amplitude	393.8 (238.4–735.0) μM 50.9 ± 12.1%	1.8 (1.2–2.6) μM 84.7 ± 3.1%
Force	> 300 μM 39.4 ± 14.9%	1.8 (1.3–2.6) μM 84.4 ± 3.1%
Duration (Half‐Width)	> 300 μM −5.9 ± 6.2%^b^	No significant effect
Muscle tone	> 300 μM −13.6 ± 9.0%^b^	No significant effect

^a^ Data are from Smith et al.^3^

^b^ Negative response indicates an increase in contractility.

### 3.2 Differential effects of CBA and 9‐phenanthrol on the excitability of isolated UBSM cells: Lack of effects of CBA on membrane potential and voltage‐step‐induced cation currents

**FIGURE 4:**
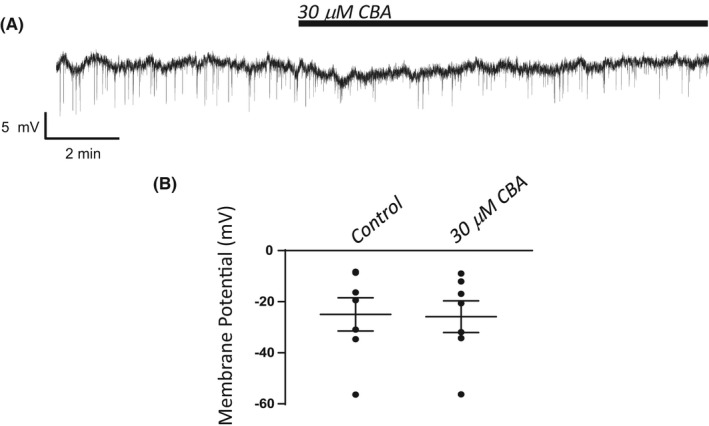
Lack of an effect by CBA on membrane potential in isolated UBSM cells. (A) Shown is a representative recording for the addition of CBA (30 μM) on the membrane potential measured in a single UBSM cell in current‐clamp (*I* = 0) mode. (B) A graph summarizing data for the control and CBA (30 μM) is displayed as individual values with means ± SEM (*n* = 7, *N* = 7).

**FIGURE 5:**
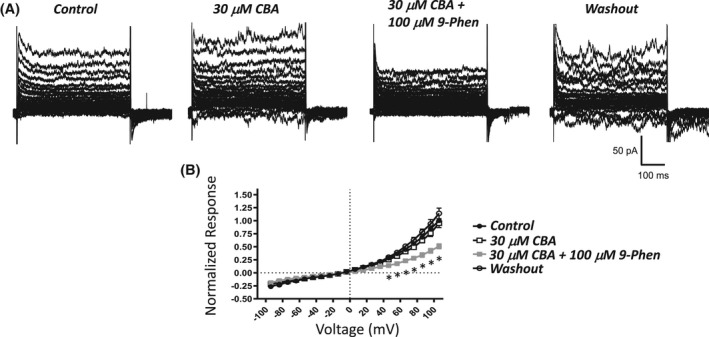
Absence of an inhibitory effect by CBA on voltage step‐induced cation currents sensitive to 9‐phenanthrol in isolated UBSM cells. (A) Depicted are representative current traces obtained for the control, 30 μM CBA, 100 μM 9‐phenanthrol (9‐Phen) added in the presence of CBA, and washout in the same UBSM cell. The UBSM cell was held at −74 mV and stepped to voltages ranging from −94 to +106 mV for 500 ms in 10 mV increments, and returned to −74 mV. (B) A summary graph illustrating normalized responses for the control, 30 μM CBA, 100 μM 9‐phenanthrol (9‐Phen) in the presence of CBA, and after washout (*n* = 3–6, *N* = 3–7). Two‐way ANOVA analysis did not reveal a statistically significant interaction for the effects of voltage and the presence of CBA (control versus 30 μM CBA, F[20100] = 0.0670, *p* = .847). A significant interaction was observed for voltage and the presence or absence of 9‐phenanthrol (control versus CBA versus 9‐Phen+CBA, *F*[40160] = 10.24, *p* < .0001); * indicates significance (*p* < .05, Sidak multiple comparison post hoc test) at each voltage for the comparisons of 9‐Phen+CBA versus both control and CBA conditions.

9‐Phenanthrol effectively hyperpolarizes UBSM cells when measured using the amphotericin B perforated patch‐clamp technique.^3^ Using the same experimental approach, we performed comparative experiments with CBA. As shown in Figure 4, CBA did not change the membrane potential of UBSM cells. The control membrane potential was −25.0 ± 6.6 mV (*n* = 7, *N* = 7), which remained comparable in the presence of 30 μM CBA, Δ −0.9 ± 0.5 mV (*n* = 7, *N* = 7, *p* > .05, paired *t*‐test). Next, the effect of CBA was examined on voltage‐step‐induced cation currents. Similar to the observation of the membrane potential, CBA did not cause any change in the voltage‐step‐induced currents (Figure 5A,B). Of note, the subsequent application of 9‐phenanthrol (100 μM) in the presence of CBA caused significant inhibition of ~50%–60% at voltages of +56 mV and above up to +106 mV (Figure 5A,B). For example, the normalized responses at +106 mV, were 1 for control, 0.95 ± 0.08 for CBA (*n* = 9, *N* = 7), and 0.41 ± 0.06 for CBA + 9‐phenanthrol (*n* = 7, *N* = 6, *p* < .01 paired t‐test vs same cells in CBA, 0.94 ± 0.10). Upon washout of CBA + 9‐phenanthrol, when experimentally possible to maintain UBSM cell recordings, the responses recovered near their control values, 1.1 ± 0.1 (*n* = 6, *N* = 6). During the course of the experiments, the cell capacitance was constantly monitored, and it did not change following the application of CBA (30 μM). The cell capacitance values were 47.5 ± 7.3 pF and 47.5 ± 7.4 pF (*n* = 9, *N* = 7) in the absence and presence of CBA, respectively. The addition of 9‐phenanthrol (100 μM) in the presence of CBA increased the cell capacitance to 53.2 ± 9.2 pF (*n* = 7, *N* = 6; *p* < .05 vs CBA‐only: 51.1 ± 10.9 pF). These experiments illustrate that the two UBSM cell excitability parameters, membrane potential, and voltage‐step‐induced currents, were unaffected by CBA.

### 3.3 Flufenamic acid (FFA), a nonselective TRPM4 channel blocker, does not reduce 9‐phenanthrol‐sensitive voltage‐step‐induced cation currents in freshly isolated UBSM cells

**FIGURE 6:**
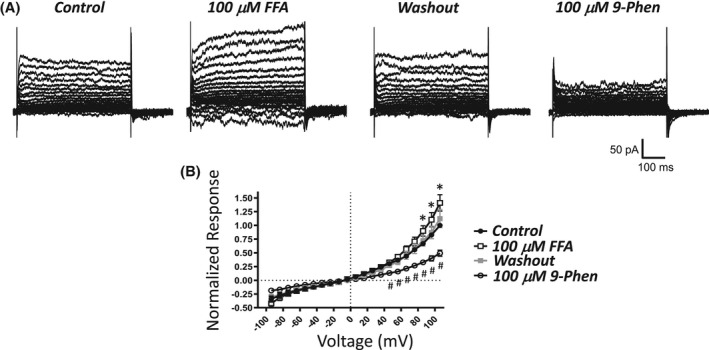
Lack of inhibitory effects of flufenamic acid, a non‐selective TRPM4 channel inhibitor, on 9‐phenanthrol‐sensitive voltage step‐induced cation currents in isolated UBSM cells. (A) Depicted are representative current traces obtained for the control, 100 μM flufenamic acid (FFA), washout of FFA/control for 9‐phenanthrol (9‐Phen), and 100 μM 9‐Phen in the same UBSM cell. The UBSM cell was held at −74 mV and stepped to voltages ranging from −94 to +106 mV for 500 ms in 10 mV increments, and returned to −74 mV. (B) A summary graph illustrates normalized responses for the control, 100 μM FFA, washout/control, and 100 μM 9‐Phen (*n* = 7, *N* = 7). Two‐way ANOVA analysis revealed a statistically significant interaction between the effects of voltage and test condition (control vs FFA vs washout/control vs 9‐Phen, F[60360] = 16.09, *p* < .0001); * and # indicate significance (*p* < .05, Sidak multiple comparison post hoc test) at each voltage for the comparisons of FFA vs control (*) and 9‐Phen vs washout (#).

FFA, albeit non‐selective, has been shown to effectively inhibit TRPM4 channels.^20^, ^26^, ^33^ Here, the effect of FFA was evaluated on voltage‐step‐induced cation currents in UBSM cells. FFA, tested at 100 μM, did not inhibit the currents. Instead, an enhancement was noted especially for voltages of +86 mV and higher. For example, at +106 mV the normalized voltage‐step‐induced current increased from 1 to 1.41 ± 0.15 (*n* = 7, *N* = 7, *p* < .05, Sidak multiple comparison post hoc test, Figure 6). The stimulatory effect of FFA was fully reversible upon washout (1.12 ± 0.16, *n* = 7, *N* = 7). The cell capacitance was not affected by FFA (*n* = 7, *N* = 7; control: 44.9 ± 7.7 pF, FFA: 45.4 ± 8.1 pF, *p* > .05, paired Student's t‐test). To further demonstrate that the specific UBSM cells tested still retained sensitivity to 9‐phenanthrol, this widely used inhibitor was subsequently added to the same UBSM cells previously evaluated for FFA following its washout. 9‐Phenanthrol effectively attenuated voltage‐step‐induced cation currents (Figure 6). In contrast to FFA, 9‐phenanthrol increased the cell capacitance in the same cells (*n* = 7, *N* = 7; control: 44.3 ± 7.2 pF, 9‐phenanthrol: 48.3 ± 7.9 pF; *p* < .02, paired *t*‐test). Collectively, voltage‐step‐induced cation currents, which were robustly blocked by 9‐phenanthrol, were not inhibited by FFA; instead, a modest current enhancement occurred.

### 3.4 BTP2, a nonselective TRPM4 channel activator, does not increase 9‐phenanthrol‐sensitive voltage‐step‐induced cation currents in isolated UBSM cells

**FIGURE 7:**
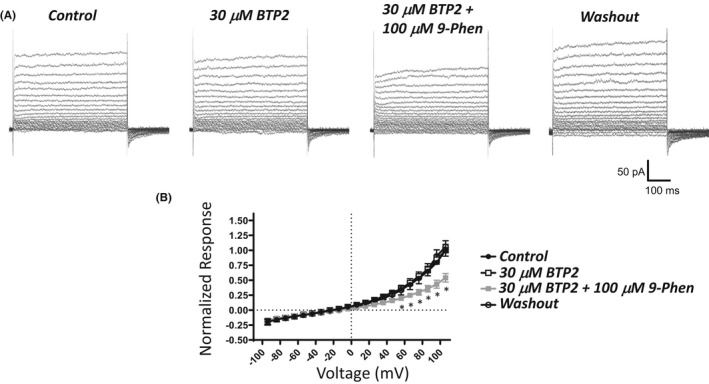
No BTP2‐induced changes in 9‐phenanthrol‐sensitive voltage step‐induced cation currents in UBSM cells. (A) Displayed are representative current traces obtained for the control, 30 μM BTP2 (or YM‐58483), 100 μM 9‐phenanthrol (9‐Phen) added in the presence of BTP2, and after washout in the same UBSM cell. The UBSM cell was held at −74 mV and stepped to voltages ranging from −94 to +106 mV for 500 ms in 10 mV increments, and returned to −74 mV. (B) A summary graph illustrates normalized responses for the control, 30 μM BTP2, 100 μM 9‐Phen in the presence of BTP2, and after washout (*n* = 3–6, *N* = 3–5). Two‐way ANOVA analysis did not reveal a statistically significant interaction for the effects of voltage and the presence of BTP2 (control versus 30 μM BTP2, F[20100] = 0.176, *p* > .9999). A significant interaction was observed for voltage and the presence or absence of 9‐Phen (control versus CBA versus 9‐Phen+BTP2, F[40160] = 10.24, *p* < .0001); * indicates significance (*p* < .05, Sidak multiple comparison post hoc test) at each voltage for the comparisons of 9‐Phen+BTP2 versus both control and BTP2 conditions.

Among the various pharmacological effects of BTP2, enhancement of TRPM4 currents has been reported.^27^ Here, the effect of BTP2 (10 μM) was evaluated on the voltage‐step‐induced cation currents. BTP2 did not alter the currents (Figure 7). The subsequent addition of 9‐phenanthrol in the presence of BTP2 decreased the currents, especially over positive voltages. Under our experimental conditions, significant current inhibitions by 9‐phenanthrol in the presence of BTP2 were observed at +56 mV and above. For example, the normalized responses at +106 mV for control, BTP2, and 9‐phenanthrol with BTP2 were 1, 1.00 ± 0.10 (*n* = 6, *N* = 5, *p* > .05, Sidak multiple comparison post hoc test), and 0.54 ± 0.07 (*n* = 5, *N* = 4, *p* < .05, Sidak multiple comparison post hoc test vs. 1.02 ± 0.12 for matched cells in BTP2), respectively. Hence, 9‐phenanthrol‐sensitive currents were not affected by BTP2. BTP2 did not change the cell capacitance (*n* = 6, *N* = 5, control: 46.4 ± 4.2 pF; BTP2: 46.3 ± 4.2 pF).

### 3.5 Depolarizing holding potentials do not increase voltage‐step‐induced cation currents in isolated UBSM cells

**FIGURE 8:**
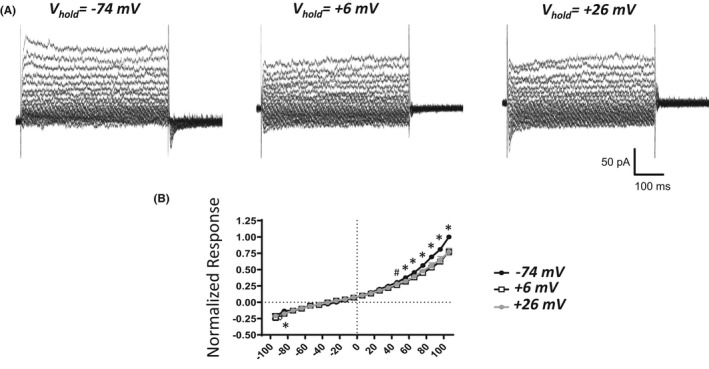
Lack of increase in voltage step‐induced cation currents with depolarizing holding potentials in isolated UBSM cells. (A) Shown are representative voltage‐step‐induced cation currents measured with the standard voltage step protocol (10 mV voltage step increments with 500 ms duration from −94 mV to +106 mV) at three different test holding potentials (V_hold_) of −74 mV, +6 mV, and + 26 mV in the same UBSM cell. (B) Summary graph displaying normalized responses versus voltage for the three test holding potentials (−74 mV, +6 mV, and + 26 mV obtained in UBSM cells [*n* = 9, *N* = 6]). Two‐way ANOVA analysis showed a significant interaction for the effects of voltage step value and test holding potential variable (−74 mV versus +6 mV versus +26 mV, F[40, 320] = 11.04, *p* < .0001); * and # indicate significance (Sidak multiple comparison post hoc test) at each voltage for −74 mV versus both +6 mV and + 26 mV (*), and − 74 mV versus +6 mV (#).

A previous study on recombinant and native TRPM4 channels reported that TRPM4 currents display dependency on the holding voltage. The TRPM4 currents show enhancement upon a strong depolarizing holding voltage.^27^ Here, the voltage‐step‐induced currents in UBSM cells were examined at the standard holding voltage V_hold_ of −74 mV – as used for all voltage‐step experiments reported so far – and compared to those determined at V_hold_ of either +6 or + 26 mV (Figure 8). Under the recording conditions, the voltage‐step‐induced currents measured at V_hold_ + 6 and + 26 mV showed reduction, especially for positive voltage steps (+46 to +106 mV), when compared to V_hold_ of −74 mV. For instance, for the voltage step to +106 mV under V_hold_ of −74, +6, and + 26 mV, the normalized responses were, respectively, 1, 0.78 ± 0.05 (*n* = 9, *N* = 6, *p* < .05, Sidak multiple comparison post hoc test), 0.78 ± 0.05 (*n* = 9, *N* = 6, *p* < .05, Sidak multiple comparison post hoc test). These experiments illustrate that in UBSM cells voltage‐step‐induced cation currents show a degree of voltage‐sensitivity based on V_hold_; depolarizing V_hold_ conditions promote reduction in the voltage‐step‐induced currents. Cell capacitance was the same for all three V_hold_ conditions (48.7 ± 4.1 pF for V_hold_ of −74 mV, +6 mV, and + 26 mV; *n* = 9, *N* = 6).

## 4 DISCUSSION

The role of TRPM4 channels in UBSM function is primarily interpreted from the inhibitory effects of 9‐phenanthrol, a widely used blocker. Here, we examined whether CBA, a novel and potent TRPM4 channel inhibitor,^20^, ^34^ could also reduce guinea pig excitability and contractility. Unlike 9‐phenanthrol, CBA displayed either no or weak inhibitory effects on UBSM contractions. In UBSM cells, CBA (30 μM) failed to replicate UBSM hyperpolarization and attenuation of voltage‐step cation currents observed for 9‐phenanthrol (this study and^3^). The TRPM4 channel inhibitor FFA^20^, ^26^, ^33^ and the activator BTP2,^27^, ^35^ did not alter the UBSM voltage‐step whole‐cell‐induced currents as expected via TRPM4 channel interaction. Collectively, TRPM4 channels in guinea pig UBSM, although expressed at the protein level,^3^, ^13^ likely determine a function distinct from excitation‐contraction regulation.

CBA displays ~3‐ to 10‐fold higher potency than 9‐phenanthrol, but comparable maximum efficacy on recombinant TRPM4 currents.^20^ We hypothesized that CBA would be more potent than 9‐phenanthrol in inhibiting UBSM contractions. Instead, CBA displayed either no or weak inhibition on spontaneous, 20 mM KCl‐induced, and EFS‐induced contractions (Figures 1, 2, 3). The modest CBA‐induced inhibition observed at the highest concentrations likely reflects non‐selectivity.^36^ In contrast, 9‐phenanthrol strongly inhibited guinea pig UBSM contractility.^3^, ^13^, ^15^ In our experiments, the maximum concentration of DMSO to dissolve CBA was 0.3% (v/v). At this concentration, the vehicle exhibits only modest effects on guinea pig UBSM contractility specifically increases up to 30% on the amplitude and muscle force and no significant effects on other contraction parameters that were associated with ~20% reduction in the tone.^15^ The effects of CBA and 9‐phenanthrol are, hence, distinct from the vehicle. CBA failed to alter the membrane potential and the voltage‐step‐induced currents (Figures 4, 5). In comparison, 9‐phenanthrol‐induced hyperpolarization and attenuated the voltage‐step‐induced currents.^3^, ^15^ CBA, hence, did not mimic the inhibitory effects of 9‐phenanthrol in guinea pig UBSM.

Several causes may underlie differences in the effects of 9‐phenanthrol and CBA. First, 9‐phenanthrol mediated effects could be due to non‐TRPM4 channel pharmacology. 9‐Phenanthrol is not very selective for TRPM4 channels as this compound also acts on TMEM16A/Ano1 Cl^−^, Ca_V_, Na_V_, K_V_, and IK channels and induces cytotoxicity.^21^, ^22^, ^23^, ^24^, ^25^ In UBSM cells, 9‐phenanthrol also increased cell capacitance, an effect independent of TRPM4 channel involvement.^15^ Unlike 9‐phenanthrol, the novel TRPM4 channel blocker CBA^20^, ^34^ and the activator BTP2^27^, ^35^ did not change the voltage‐step‐induced cation currents in UBSM cells (Figures 5 and 7). FFA, another non‐selective TRPM4 channel blocker,^20^, ^26^, ^33^ did not attenuate the currents but induced either no change or increase at very positive voltages (Figure 8). The enhancing effect of FFA may be due to the activation of TRPA1 channels.^37^ Among the tested TRPM4 channel modulators only 9‐phenanthrol displayed robust inhibitory efficacy on UBSM electrical and mechanical activities. Second, differential effects of 9‐phenanthrol and CBA may be potentially explained by species differences. At present, the full sequence of the guinea pig TRPM4 channel has not yet been cloned, precluding its expression and thus investigation. In contrast, both 9‐phenanthrol and CBA fully blocked recombinant human TRPM4 channels when expressed in HEK293 and tsa201 cells.^20^, ^21^ For mouse TRPM4 channels, contradictory findings exist. CBA in excised patches of mouse recombinant TRPM4 channels either did not affect the cation currents or caused potentiation when applied extracellularly or intracellularly, respectively.^21^ 9‐Phenanthrol applied extracellularly but not intracellularly blocked TRPM4 currents.^21^ This orientation‐specificity for 9‐phenanthrol was surprising given high hydrophobicity of 9‐phenanthrol (xLogP of 3.6, see PubChem), and the ease with which it is expected to cross the cell membrane. Conversely, native mouse TRPM4 currents in pancreatic acinar cells were inhibited by CBA and 9‐phenanthrol.^38^ Future studies are needed to establish how CBA affects mouse and guinea pig TRPM4 channels. Third, TRPM4 channels in UBSM exist in a configuration state regardless of species that exhibit a unique pharmacological profile: sensitivity to 9‐phenanthrol, insensitivity to CBA and FFA, and only partial inhibition by glibenclamide, another non‐selective TRPM4 channel blocker.^15^ Among these explanations, we favor the first that the effects of 9‐phenanthrol in UBSM are due to a non‐TRPM4 channel interaction.

Other confounding factors in our electrophysiological experiments are the temperature‐dependency of TRPM4 channels and dependency on intracellular Ca^2+^.^39^ Half voltage activation constant for TRPM4 currents shifted ~ −50 mV when changed from 20 to 31°C, and the channel opening required high Ca^2+^ (>10–100 μM).^39^ Our electrophysiological recordings were conducted at room temperature and under condition where intracellular Ca^2+^ levels could not be controlled experimentally. We acknowledge these limitations. TRPM4 currents, however, have been recorded in cerebral vascular smooth muscle cells using the same experimental approach as utilized here.^40^, ^41^ Recombinant TRPM4 currents have been routinely measured at room temperature.^9^, ^20^ If temperature played a role, then there should have been a discrepancy observed between electrophysiological and contractile responses for CBA measured, respectively, at ~22°C and 37°C. This was not observed. CBA lacked robust inhibitory responses. Contraction experiments also illuminate the issue of elevated intracellular Ca^2+^. Both 20 mM KCl‐induced contractions and EFS‐induced contractions elevate intracellular Ca^2+^ levels, and yet CBA efficacy remained comparable and weak for all three contractility protocols (Figures 1, 2, 3). Of note, for 20 mM KCl‐induced contractions, we observed an enhancement in the frequency for the highest CBA concentrations, most likely due to non‐selectivity. As CBA has been recently shown to inhibit transient outward K^+^ current and late Na^+^ current in ventricular myocytes,^36^ this compound may lack optimal selectivity.

Given the issues with the currently available TRPM4 channel pharmacological tools, determining the roles of TRPM4 channel function in UBSM will require additional approaches. The utility of genetic animal models, whether global or inducible conditional, will be extremely useful as has been the case for elucidating the roles of TRPM4 channels in the heart, taste buds, brain, and pancreas.^38^, ^42^, ^43^, ^44^, ^45^, ^46^ Unfortunately, the specific TRPM4 knock‐out mouse or rat strains have not yet been made available to us. We look forward to these prospective studies providing further insights.

As TRPM4 channels are robustly expressed in UBSM cells and tissues of humans, rats, guinea pigs, and mice,^3^, ^4^, ^5^, ^7^, ^12^ a question arises as to their overall physiological role in the urinary bladder. Our current study provides evidence that they may not directly regulate UBSM excitability or contractility. For non‐smooth muscle cells, TRPM4 channels have been implicated in the regulation of hypertrophy, cell growth, proliferation, migration, and cell differentiation promoting fibrosis.^45^, ^47^, ^48^, ^49^ Additionally, TRPM4 channels form interacting complexes with TRPC3 and SUR1 subunits as well as cytoskeletal adhesion proteins affecting their properties,^50^, ^51^, ^52^ although in healthy UBSM cells TRPM4‐SUR1 complexes are unlikely to be functional.^15^ TRPM4 channels may play one of these roles in UBSM cells. A recent report found a time‐dependent upregulation of TRPM4 channel expression in the bladder of a spinal cord injury mouse model suggesting a contribution of TRPM4 channels to post‐injury healing.^7^ TRPM4 channel protein expression also changed during maturation from youth to adulthood in guinea pigs for both intracellular and cell surface expression perhaps related to the putative non‐excitatory, non‐contractile regulatory role.^13^ Additional mechanistic studies are, therefore, needed to unravel the functional role of TRPM4 channels in UBSM.

In summary, the novel TRPM4 channel inhibitor CBA did not mimic the inhibitory effects of the classical and non‐selective inhibitor 9‐phenanthrol on UBSM electrical and mechanical activities. Two other TRPM4 channel modulators FFA and BTP2 did not affect UBSM cation currents as expected for the TRPM4 channel engagement. Our study, hence, suggests that TRPM4 channels, although expressed in UBSM, play an additional role (e.g., in cell proliferation or under pathophysiological conditions) rather than regulation of excitability and contractility.

## AUTHOR CONTRIBUTIONS

*Participated in research design*: Malysz, Maxwell, and Petkov. *Conducted experiments*: Malysz and Maxwell. *Performed data analyses*: Malysz and Maxwell. *Wrote or contributed to the writing of the manuscript*: Malysz, Maxwell, and Petkov.

## FUNDING INFORMATION

This publication was supported by the National Institute of Diabetes and Digestive and Kidney Diseases under Award Numbers R01 DK‐106964 and P20 DK‐123971. The content is solely the responsibility of the authors and does not necessarily represent the official views of the National Institutes of Health.

## DISCLOSURE

The authors declare no conflict of interest.

## ETHICS STATEMENT

Experiments were conducted in accordance with the Animal Use Protocols Nos. 17‐075 and 20‐019 reviewed and approved by the University of Tennessee Health Science Center (Memphis, TN).

## Data Availability

The data supporting the findings of this study are available from the corresponding authors upon request.

## References

[1] Andersson KE , Arner A . Urinary bladder contraction and relaxation: physiology and pathophysiology. Physiol Rev. 2004;84:935‐986. 15269341 10.1152/physrev.00038.2003

[2] Malysz J , Petkov GV . Urinary bladder smooth muscle ion channels: expression, function, and regulation in health and disease. Am J Physiol Renal Physiol. 2020;319:F257‐F283. 32628539 10.1152/ajprenal.00048.2020 PMC7473901

[3] Smith AC , Hristov KL , Cheng Q , et al. Novel role for the transient potential receptor melastatin 4 channel in Guinea pig detrusor smooth muscle physiology. Am J Physiol Cell Physiol. 2013;304:C467‐C477. 23302778 10.1152/ajpcell.00169.2012 PMC3602646

[4] Smith AC , Parajuli SP , Hristov KL , et al. TRPM4 channel: a new player in urinary bladder smooth muscle function in rats. Am J Physiol Renal Physiol. 2013;304:F918‐F929. 23283997 10.1152/ajprenal.00417.2012 PMC3625855

[5] Hristov KL , Smith AC , Parajuli SP , Malysz J , Rovner ES , Petkov GV . Novel regulatory mechanism in human urinary bladder: central role of transient receptor potential melastatin 4 channels in detrusor smooth muscle function. Am J Physiol Cell Physiol. 2016;310:C600‐C611. 26791488 10.1152/ajpcell.00270.2015 PMC4824161

[6] Alom F , Matsuyama H , Nagano H , Fujikawa S , Tanahashi Y , Unno T . Involvement of transient receptor potential melastatin 4 channels in the resting membrane potential setting and cholinergic contractile responses in mouse detrusor and ileal smooth muscles. J Vet Med Sci. 2019;81:217‐228. 30518701 10.1292/jvms.18-0631 PMC6395210

[7] Kullmann FA , Beckel JM , McDonnell B , et al. Involvement of TRPM4 in detrusor overactivity following spinal cord transection in mice. Naunyn Schmiedebergs Arch Pharmacol. 2018;391:1191‐1202. 30054681 10.1007/s00210-018-1542-0 PMC6186176

[8] Nilius B , Owsianik G . The transient receptor potential family of ion channels. Genome Biol. 2011;12:218. 21401968 10.1186/gb-2011-12-3-218 PMC3129667

[9] Nilius B , Prenen J , Droogmans G , et al. Voltage dependence of the Ca^2+^‐activated cation channel TRPM4. J Biol Chem. 2003;278:30813‐30820. 12799367 10.1074/jbc.M305127200

[10] Wang C , Naruse K , Takahashi K . Role of the TRPM4 channel in cardiovascular physiology and pathophysiology. Cell. 2018;7:E62. 10.3390/cells7060062 PMC6025450 29914130

[11] Earley S . TRPM4 channels in smooth muscle function. Pflugers Arch. 2013;465:1223‐1231. 23443854 10.1007/s00424-013-1250-z PMC3686874

[12] Parajuli SP , Hristov KL , Sullivan MN , et al. Control of urinary bladder smooth muscle excitability by the TRPM4 channel modulator 9‐phenanthrol. Channels (Austin). 2013;7:537‐540. 24037125 10.4161/chan.26289 PMC4042489

[13] Maxwell SE , Leo MD , Malysz J , Petkov GV . Age‐dependent decrease in TRPM4 channel expression but not trafficking alters urinary bladder smooth muscle contractility. Physiol Rep. 2021;9:e14754. 33625779 10.14814/phy2.14754 PMC7903938

[14] Guinamard R , Hof T , Del Negro CA . The TRPM4 channel inhibitor 9‐phenanthrol. Br J Pharmacol. 2014;171:1600‐1613. 24433510 10.1111/bph.12582 PMC3966741

[15] Malysz J , Maxwell SE , Yarotskyy V , Petkov GV . TRPM4 channel inhibitors 9‐phenanthrol and glibenclamide differentially decrease Guinea pig detrusor smooth muscle whole‐cell cation currents and phasic contractions. Am J Physiol Cell Physiol. 2020;318:C406‐C421. 31851526 10.1152/ajpcell.00055.2019 PMC7052614

[16] Barbet G , Demion M , Moura IC , et al. The calcium‐activated nonselective cation channel TRPM4 is essential for the migration but not the maturation of dendritic cells. Nat Immunol. 2008;9:1148‐1156. 18758465 10.1038/ni.1648 PMC2956271

[17] Cheng H , Beck A , Launay P , et al. TRPM4 controls insulin secretion in pancreatic beta‐cells. Cell Calcium. 2007;41:51‐61. 16806463 10.1016/j.ceca.2006.04.032 PMC5663640

[18] Serafini N , Dahdah A , Barbet G , et al. The TRPM4 channel controls monocyte and macrophage, but not neutrophil, function for survival in sepsis. J Immunol. 2012;189:3689‐3699. 22933633 10.4049/jimmunol.1102969

[19] Grand T , Demion M , Norez C , et al. 9‐phenanthrol inhibits human TRPM4 but not TRPM5 cationic channels. Br J Pharmacol. 2008;153:1697‐1705. 18297105 10.1038/bjp.2008.38 PMC2438271

[20] Ozhathil LC , Delalande C , Bianchi B , et al. Identification of potent and selective small molecule inhibitors of the cation channel TRPM4. Br J Pharmacol. 2018;175:2504‐2519. 29579323 10.1111/bph.14220 PMC6002741

[21] Arullampalam P , Preti B , Ross‐Kaschitza D , Lochner M , Rougier JS , Abriel H . Species‐specific effects of cation channel TRPM4 small‐molecule inhibitors. Front Pharmacol. 2021;12:712354. 34335274 10.3389/fphar.2021.712354 PMC8321095

[22] Burris SK , Wang Q , Bulley S , Neeb ZP , Jaggar JH . 9‐phenanthrol inhibits recombinant and arterial myocyte TMEM16A channels. Br J Pharmacol. 2015;172:2459‐2468. 25573456 10.1111/bph.13077 PMC4409899

[23] Hou JW , Fei YD , Li W , et al. The transient receptor potential melastatin 4 channel inhibitor 9‐phenanthrol modulates cardiac sodium channel. Br J Pharmacol. 2018;175:4325‐4337. 30153324 10.1111/bph.14490 PMC6240128

[24] Simard C , Salle L , Rouet R , Guinamard R . Transient receptor potential melastatin 4 inhibitor 9‐phenanthrol abolishes arrhythmias induced by hypoxia and re‐oxygenation in mouse ventricle. Br J Pharmacol. 2012;165:2354‐2364. 22014185 10.1111/j.1476-5381.2011.01715.x PMC3413868

[25] Veress R , Baranyai D , Hegyi B , et al. Transient receptor potential melastatin 4 channel inhibitor 9‐phenanthrol inhibits K^+^ but not Ca^2+^ currents in canine ventricular myocytes. Can J Physiol Pharmacol. 2018;96:1022‐1029. 29806985 10.1139/cjpp-2018-0049

[26] Amarouch MY , Syam N , Abriel H . Biochemical, single‐channel, whole‐cell patch clamp, and pharmacological analyses of endogenous TRPM4 channels in HEK293 cells. Neurosci Lett. 2013;541:105‐110. 23428507 10.1016/j.neulet.2013.02.011

[27] Takezawa R , Cheng H , Beck A , et al. A pyrazole derivative potently inhibits lymphocyte Ca^2+^ influx and cytokine production by facilitating transient receptor potential melastatin 4 channel activity. Mol Pharmacol. 2006;69:1413‐1420. 16407466 10.1124/mol.105.021154

[28] Malysz J , Rovner ES , Wake R , Petkov GV . Preparation and utilization of freshly isolated human detrusor smooth muscle cells for characterization of 9‐phenanthrol‐sensitive cation currents. J Vis Exp. 2020. doi: 10.3791/59884 PMC7489995 32065126

[29] Malysz J , Buckner SA , Daza AV , Milicic I , Perez‐Medrano A , Gopalakrishnan M . Functional characterization of large conductance calcium‐activated K^+^ channel openers in bladder and vascular smooth muscle. Naunyn Schmiedebergs Arch Pharmacol. 2004;369:481‐489. 15095032 10.1007/s00210-004-0920-y

[30] Shiga KI , Hirano K , Nishimura J , Niiro N , Naito S , Kanaide H . Dimethyl sulphoxide relaxes rabbit detrusor muscle by decreasing the Ca^2+^ sensitivity of the contractile apparatus. Br J Pharmacol. 2007;151:1014‐1024. 17549043 10.1038/sj.bjp.0707317 PMC2042939

[31] Harding SD , Sharman JL , Faccenda E , Southan C , Pawson AJ , Ireland S , Gray AJG , Bruce L , Alexander SPH , Anderton S , Bryant C , Davenport AP , Doerig C , Fabbro D , Levi‐Schaffer F , Spedding M , Davies JA , NC‐IUPHAR (2018). The IUPHAR/BPS guide to pharmacology in 2019: updates and expansion to encompass the new guide to immunopharmacology. Nucleic Acids Res 46: D1091‐1106. 10.1093/nar/gkx1121 29149325 PMC5753190

[32] Alexander SPH , Kelly E , Mathie A , et al. The concise guide to pharmacology 2019/20: introduction and other protein targets. Br J Pharmacol. 2019;176(S1):S1‐S10. 31710719 10.1111/bph.14747 PMC6844537

[33] Constantine M , Liew CK , Lo V , et al. Heterologously‐expressed and liposome‐reconstituted human transient receptor potential melastatin 4 channel (TRPM4) is a functional tetramer. Sci Rep. 2016;6:19352. 26785754 10.1038/srep19352 PMC4726259

[34] Delalande C , Awale M , Rubin M , et al. Optimizing TRPM4 inhibitors in the MHFP6 chemical space. Eur J Med Chem. 2019;166:167‐177. 30708257 10.1016/j.ejmech.2019.01.048

[35] Riquelme D , Silva I , Philp AM , et al. Subcellular localization and activity of TRPM4 in medial prefrontal cortex layer 2/3. Front Cell Neurosci. 2018;12:12. 29440991 10.3389/fncel.2018.00012 PMC5797675

[36] Dienes C , Hézső T , Kiss DZ , et al. Electrophysiological effects of the transient receptor potential melastatin 4 channel inhibitor (4‐chloro‐2‐[2‐chlorophenoxy]acetamido)benzoic acid (CBA) in canine left ventricular cardiomyocytes. Int J Mol Sci. 2021;22:9499. 34502410 10.3390/ijms22179499 PMC8430982

[37] Hu H , Tian J , Zhu Y , et al. Activation of TRPA1 channels by fenamate nonsteroidal anti‐inflammatory drugs. Pflugers Arch. 2010;459:579‐592. 19888597 10.1007/s00424-009-0749-9 PMC2828537

[38] Diszhazi G , Magyar ZE , Lisztes E , et al. TRPM4 links calcium signaling to membrane potential in pancreatic acinar cells. J Biol Chem. 2021;297:101015. 34329682 10.1016/j.jbc.2021.101015 PMC8371206

[39] Talavera K , Yasumatsu K , Voets T , et al. Heat activation of TRPM5 underlies thermal sensitivity of sweet taste. Nature. 2005;438:1022‐1025. 16355226 10.1038/nature04248

[40] Gonzales AL , Amberg GC , Earley S . Ca^2+^ release from the sarcoplasmic reticulum is required for sustained TRPM4 activity in cerebral artery smooth muscle cells. Am J Physiol Cell Physiol. 2010;299:C279‐C288. 20427713 10.1152/ajpcell.00550.2009 PMC2928641

[41] Gonzales AL , Earley S . Endogenous cytosolic Ca^2+^ buffering is necessary for TRPM4 activity in cerebral artery smooth muscle cells. Cell Calcium. 2012;51:82‐93. 22153976 10.1016/j.ceca.2011.11.004 PMC3265659

[42] Bovet‐Carmona M , Menigoz A , Pinto S , et al. Disentangling the role of TRPM4 in hippocampus‐dependent plasticity and learning: an electrophysiological, behavioral and FMRI approach. Brain Struct Funct. 2018;223:3557‐3576. 29971514 10.1007/s00429-018-1706-1

[43] Demion M , Thireau J , Gueffier M , et al. Trpm4 gene invalidation leads to cardiac hypertrophy and electrophysiological alterations. PLoS One. 2014;9:e115256. 25531103 10.1371/journal.pone.0115256 PMC4274076

[44] Dutta Banik D , Martin LE , Freichel M , Torregrossa AM , Medler KF . TRPM4 and TRPM5 are both required for normal signaling in taste receptor cells. Proc Natl Acad Sci U S A. 2018;115:E772‐E781. 29311301 10.1073/pnas.1718802115 PMC5789955

[45] Frede W , Medert R , Poth T , et al. TRPM4 modulates right ventricular remodeling under pressure load accompanied with decreased expression level. J Card Fail. 2020;26:599‐609. 32147520 10.1016/j.cardfail.2020.02.006

[46] Hedon C , Lambert K , Chakouri N , et al. New role of TRPM4 channel in the cardiac excitation‐contraction coupling in response to physiological and pathological hypertrophy in mouse. Prog Biophys Mol Biol. 2021;159:105‐117. 33031824 10.1016/j.pbiomolbio.2020.09.006

[47] Borgstrom A , Peinelt C , Stoklosa P . TRPM4 in cancer‐a new potential drug target. Biomolecules. 2021;11:229. 33562811 10.3390/biom11020229 PMC7914809

[48] Simard C , Magaud C , Adjlane R , et al. TRPM4 non‐selective cation channel in human atrial fibroblast growth. Pflugers Arch. 2020;472:1719‐1732. 33047172 10.1007/s00424-020-02476-0

[49] Wang F , Wu P , Gong S , et al. Aberrant TRPM4 expression in MLL‐rearranged acute myeloid leukemia and its blockade induces cell cycle arrest via AKT/GLI1/cyclin D1 pathway. Cell Signal. 2020;72:109643. 32320859 10.1016/j.cellsig.2020.109643

[50] Caceres M , Ortiz L , Recabarren T , et al. TRPM4 is a novel component of the adhesome required for focal adhesion disassembly, migration and contractility. PLoS One. 2015;10:e0130540. 26110647 10.1371/journal.pone.0130540 PMC4482413

[51] Cho CH , Lee YS , Kim E , Hwang EM , Park JY . Physiological functions of the TRPM4 channels via protein interactions. BMB Rep. 2015;48:1‐5. 25441424 10.5483/BMBRep.2015.48.1.252 PMC4345635

[52] Park JY , Hwang EM , Yarishkin O , et al. TRPM4b channel suppresses store‐operated Ca^2+^ entry by a novel protein‐protein interaction with the TRPC3 channel. Biochem Biophys Res Commun. 2008;368:677‐683. 18262493 10.1016/j.bbrc.2008.01.153

